# Vacuum bell therapy for pectus excavatum: a retrospective study

**DOI:** 10.1186/s12887-024-04615-3

**Published:** 2024-03-09

**Authors:** Weixuan Lei, Mengqi Shao, Yan Hu, Jieming Cao, Wei Han, Ruoyao Wang, Quanming Fei, Jian Zou, Junqi Yi, Zheyu Cheng, Wenliang Liu

**Affiliations:** 1 Department of Thoracic Surgery, The Second Xiangya Hospital of Central South University, Changsha, 410000 China; 2 Hunan Key Laboratory of Early Diagnosis and Precise Treatment of Lung Cancer, The Second Xiangya Hospital of Central South University, Changsha, China

**Keywords:** Pectus excavatum, Non-invasive therapy, Vacuum bell therapy

## Abstract

**Background:**

Pectus excavatum, the most common chest wall deformity, is frequently treated with Nuss procedure. Here we will describe non-invasive procedure and analyze the variables associated vacuum bell therapy for patients with pectus excavatum.

**Methods:**

Retrospective case–control study in a single center between July 2018 and February 2022, including patients with pectus excavatum treated with vacuum bell. Follow-up was continued to September 2022. The Haller index and Correction index was calculated before and after treatment to analysis the effectiveness of vacuum bell therapy.

**Results:**

There were 98 patients enrolled in the treatment group, with 72 available for analysis, and the follow-up period ranged from 1.1 to 4.4 years (mean 3.3 years). When analyzing with the Haller Index, 18 patients (25.0%) showed excellent correction, 13 patients (18.1%) achieved good correction, and 4 patients (5.6%) had fair correction. The remaining patients had a poor outcome. Characteristics predicting a non-poor prognosis included initial age ≤ 11 years (OR = 3.94, *p* = 0.013) and patients with use over 24 consecutive months (OR = 3.95, *p* = 0.013). A total of 9 patients (12.5%) achieved a CI reduction below 10. Patients who started vacuum bell therapy at age > 11 had significantly less change compared to those who started at age ≤ 11 (*P* < 0.05). Complications included chest pain (5.6%), swollen skin (6.9%), chest tightness (1.4%) and erythema (15.3%).

**Conclusions:**

A certain percentage of patients with pectus excavatum can achieve excellent correction when treated with pectus excavatum therapy. Variables predicting better outcome including initial age ≤ 11 years both in HI and CI and vacuum bell use over 24 consecutive months in HI. In summary, pectus excavatum is an emerging non-invasive therapy for pectus excavatum and will be widely performed in a certain group of patients.

**Supplementary Information:**

The online version contains supplementary material available at 10.1186/s12887-024-04615-3.

## Introduction

Pectus excavatum (PE) is a deformity of the chest wall characterized by the posterior displacement of the sternum and adjacent ribs, resulting in a concave appearance of the chest wall that resembles a funnel or boat shape. This condition is often accompanied by other abnormalities such as rib flare or scoliosis [1–5]. For over a century, surgical treatment for pectus excavatum has been utilized, employing open procedures such as rib resection and sternotomy [6]. However, with the advent of minimally invasive surgeries, such as the Nuss procedure [7–9], surgical trauma associated with pectus excavatum has decreased while corrective outcomes have improved [6, 7].

Although the idea of applying suction to the chest wall to elevate the sternum was first described over a century ago, the refinement of this method has taken place in the last 20 years [2, 6–12]. Schier et al. were the first to describe the use of the vacuum bell as an adjunct during the Nuss procedure to elevate the sternum when creating the substernal tunnel [10]. The use of the vacuum bell as an alternative to operative management has been extensively reported on by Haecker, et al. in Switzerland [7–9]. They also aptly noted that the growth spurt during puberty likely adversely influences vacuum bell outcomes owing to the typical progression of pectus depth and severity [8]. Lopez et al. also recently reported that patients less than 18 years old had better outcomes [13]. However, it remains unclear from these studies specifically which patients are most likely to achieve an excellent correction of pectus excavatum with vacuum bell therapy.

Our study aims to identify variables associated with an excellent correction and thus help optimize patient selection. Vacuum bell therapy is an alternative to the traditional Nuss procedure non-invasive therapy.

## Methods

### Ethics statement

The research presented here has been performed in accordance with the Declaration of Helsinki, and cases enrolled in this study were collected and approved by the ethical review committees from the second Xiangya hospital, Central South University, China (Ethical approval number: 2019–S423). Written informed consent was obtained from all the participants and parents or legal guardians, and any questions answered prior to participation in this study. All methods were carried out in accordance with relevant guidelines and regulations.

### Patients

Selected patient data were retrospectively collected between February 2018 and January 2022. The inclusion criteria were: mild thoracic malformations, moderate and severe thoracic malformations with no desire for surgery, and recurrent pectus excavatum after Nuss repair [14]. The exclusion criteria were: < 3 years-old, bone diseases (such as osteogenesis imperfecta, osteoporosis, Gleason's disease), vascular diseases (such as Marfan's disease, aortic aneurysm, aortic root dilatation), clotting diseases (such as hemophilia, thrombocytopenia), and other heart diseases.

Adapting to the use of vacuum bell typically takes around five weeks, gradually increasing the time and negative pressure applied. During the first week, it is recommended to use them for 30 min each in the morning and evening, with the pressure controlled to lift the indentation 1/4 to 1/2. By the fifth week, the duration should reach 2.5 h per day, and the negative pressure should be enough to flatten the indentation. Adults need to double the usage time, and patients may increase the usage time and frequency based on their own conditions, but each use should not exceed 2 h, with a 10-min interval between uses. The negative pressure should not exceed a certain limit, and children under 6 years old should not exceed 8 kPa, while those under 18 should be kept within 1-15 kPa [15, 16].

### Data collection

CT scanning was performed for patients appointed to the investigation by a physician. During the examination, the patient was in the typical chest scanning position, breathing withheld. Routine clinical follow-ups were conducted in all patients every six to twelve months with clinical and CT scanning assessments.

The Haller index, symmetry, and shape of the patients' chest wall were documented. Pulmonary function studies and cardiac ultrasound were optional. The average minutes applied of vacuum bell per day and the duration of therapy were reported by included patients.

In assessment of chest asymmetry, left and right sides chest depth ratio was calculated, according to the following equation: Asymmetry index = 1—(left side chest depth ratio / right side chest depth ratio). The chest wall is considered "asymmetric" without falling within the range of ± 0.05 for the asymmetry index [17]. The Haller index (HI) is calculated by dividing the transverse diameter of the chest by the anterior–posterior distance on CT of the chest on the axial slice that demonstrates the smallest distance between the anterior surface of the vertebral body and the posterior surface of the sternum [13, 18]. The Correction Index (CI) involves measuring two distances after drawing a horizontal line across the anterior spine. It is obtained by dividing the difference between the minimum distance from the posterior sternum to the anterior spine and the maximum distance from the line on the anterior spine to the inner margin of the most anterior part of the chest by the maximum chest prominence (longer measurement). A CI greater than 10 indicates that over 10% of the chest depth between the anterior chest and anterior spine is centrally depressed, in line with the definition of pectus excavatum [14].

### Statistical methods

The standard HI index of Nuss operation was greater than 3.25 [7, 11, 19–21], and this was used as the baseline HI to calculate the Percentage Correction of the treatment group as follows:

$$Pecentage\ Correction=\frac{Patient\ Initial\ HI-Patient\ Current\ HI}{Patient\ Intial\ HI-Baseline\ HI}\times 100\%$$

, where Baseline HI = 3.25.

This formula provides an objective measurement that describes any patient with a HI of 3.25 or less as being 100% corrected effectively. The results were divided into four categories based on the Percentage Correction as follows: Excellent ≥ 100%; Good 99%–67%; Fair 66%–34%; Poor ≤ 33%.

Descriptive statistics and frequencies are reported for primary variables. Bivariate analysis was performed via a series of binary logistic regressions to identify variables that separately predicted different outcome [22]. All analysis were conducted using SPSS version 22.0 statistical software.

## Results

**Fig. 1:**
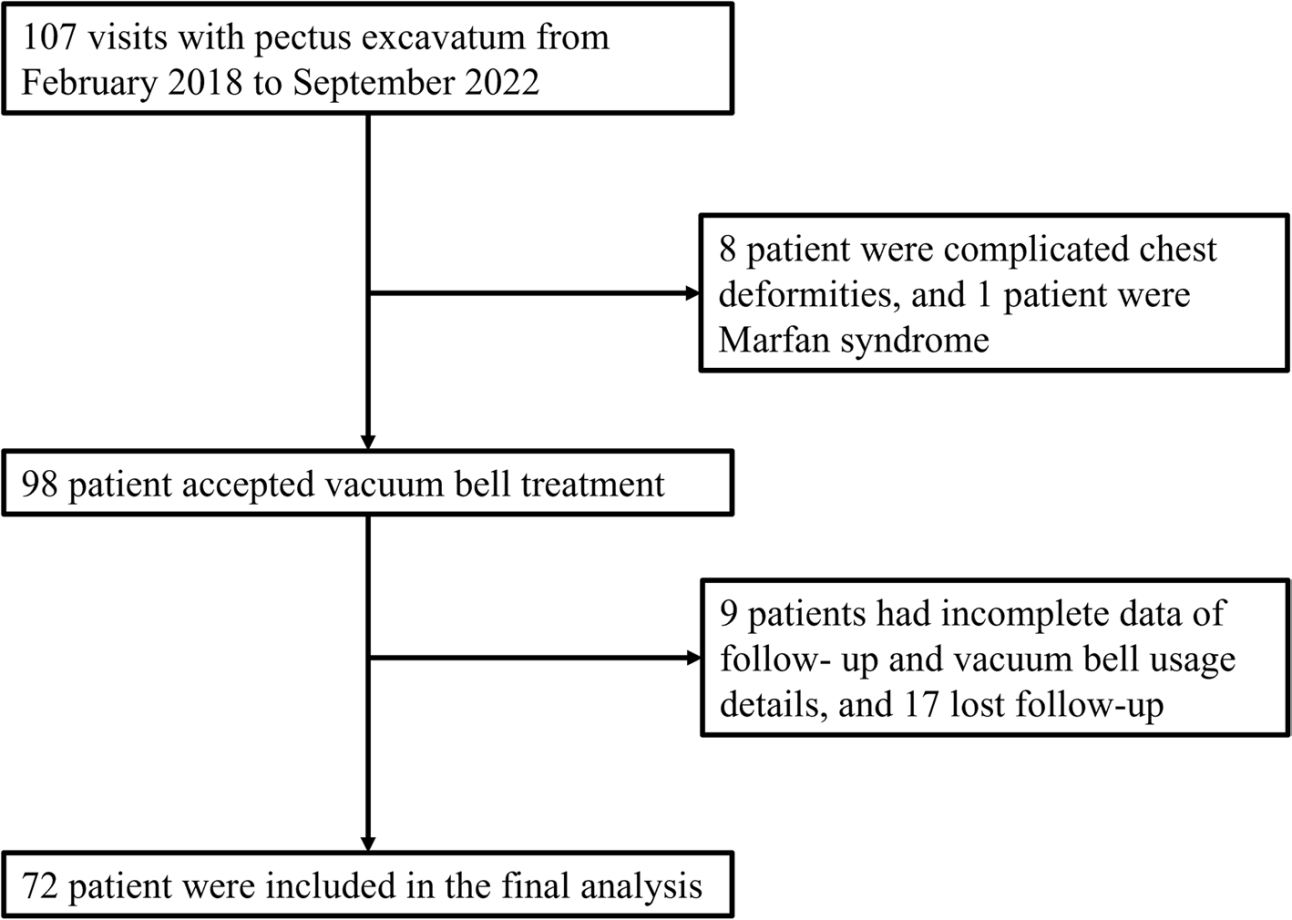
Study flow chart

**Fig. 2:**
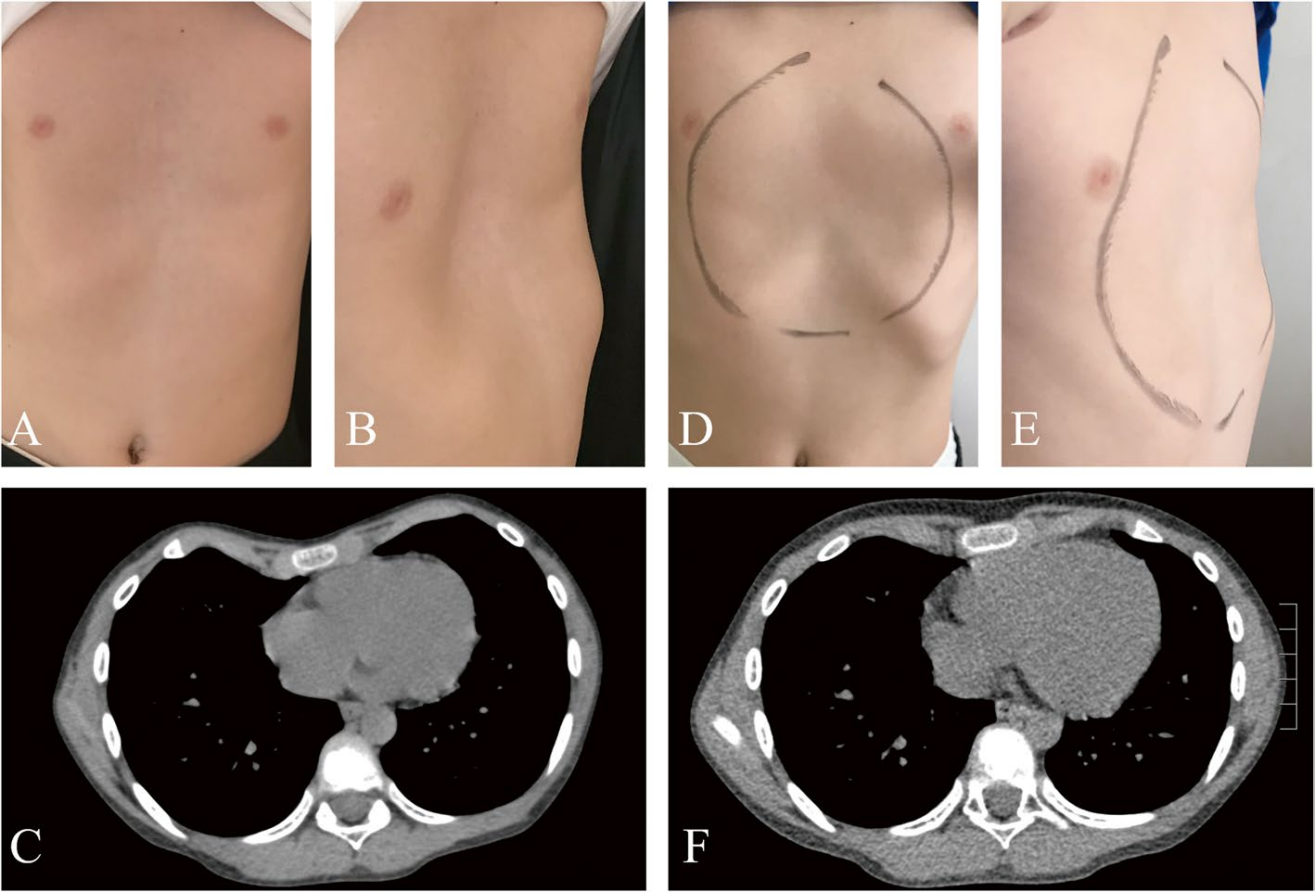
Evaluation of treatment outcomes after vacuum bell application. Pictures and chest computed tomography (CT) were taken from a 9-year-old male patient just before starting treatment (**A**, **B**, **C**) and after vacuum bell therapy (**D**, **E**, **F**). **A** Anterior view before vacuum bell application; **B** Lateral view before vacuum bell application; **C** Chest computed tomography taken before starting therapy; **D** Anterior view after treatment; **E** Lateral view after treatment; **F** Chest computed tomography taken after treatment

**Fig. 3:**
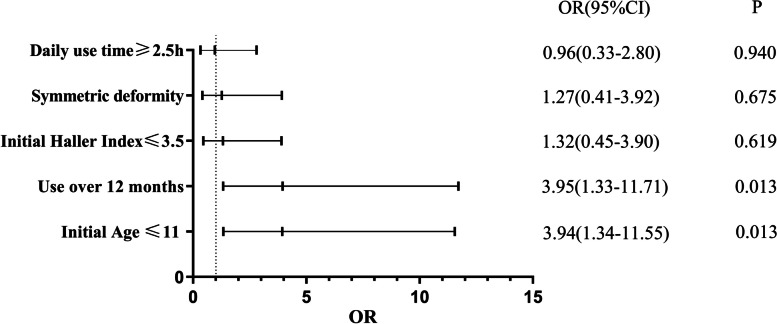
Characteristics associated with different outcome. Multivariable logistic regression analysis of the prediction of excellent, good and fair outcomes based on demographics and vacuum bell usage details in patients

From Feb. 2018 to Sep. 2022, a total of 107 consecutive patients with pectus excavatum were admitted to our outpatient clinic. Among them, 8 patients had complicated chest deformities, and 1 patient had Marfan syndrome, without undergoing cupping therapy. Out of the 98 individuals undergoing vacuum bell therapy, 26 patients (26.53%) were excluded from the analysis: 17 lost to follow-up, 5 discontinued use, and 4 lacked sufficient documentation. 72 patients were available for final evaluation, of which 57 (79.17%) were male (Fig. 1). The final evaluated patients had a mean age of 11.0 ± 4.8 years old, ranging from 3 to 24. The cohort included 9 cases of recurrence in patients who underwent a Nuss procedure. Only 5 patients in the treatment group were 18 years of age and older. The initial average Haller index in the treatment group was 3.73 ± 1.01, and the final average Haller index was 3.49 ± 1.02 (Table 1). A total of 18 (25.0%) patients demonstrated an excellent correction (Final HI ≤ 3.25), while 13 (18.1%) patients achieved a good correction (Fig. 2). Additionally, 4 patients (5.6%) exhibited a fair correction. The remaining patients experienced a poor outcome (Table 2). Patient characteristics predictive of excellent, good and fair outcome included initial age ≤ 11 years (OR = 3.94, *p* = 0.013), and use of the vacuum ball for over 24 consecutive months (OR = 6.70, *p* = 0.014). In our study, there was no clear evidence of associations between a symmetric deformity (OR = 1.27, *p* = 0.675), initial Haller index ≤ 3.5 (OR = 1.32, *p* = 0.619), and daily use time ≥ 150 min (OR = 0.96, *p* = 0.940) with improved outcomes (Fig. 3).

**Table 1:** Included patients’ characteristics

Clinical characteristic	Total (*N* = 72)
Age (years)	
Range	3–24
mean	11.02
Male/female	57/15
Symmetric deformity(yes)	48(66.7%)
Initial Haller index	3.73 ± 1.01
Final Haller index	3.49 ± 1.02
Initial Correction index	27 ± 12
Final Correction index	23 ± 13
Post- Nuss surgery	9(12.5%)

**Table 2:** Outcomes of vacuum bell therapy

Outcome	Total (*N* = 72)
Excellent (≥ 100%)	18 (25.0%)
Good (99%-67%)	13 (18.1%)
Fair (66%-34%)	4 (5.6%)
poor (≤ 33%)	37 (51.4%)

The initial average Correction index in the treatment group was 27.35 ± 12.31, and the final average Correction index was 23.22 ± 13.47 (Table 1), and decreased by 4.12 ± 7.96 at the latest follow up. A total of 9 patients (12.5%) achieved a CI reduction below 10. Analysis demonstrated that the patients who started vacuum bell therapy after the age of 11 actually had significantly less change in Correction index when compared to those who started under 11 (*P* < 0.05). In our study, there were no clear associations observed between Gender (*p* = 0.151), daily use time ≥ 150 min (*p* = 0.058), use of the vacuum bell for over 24 consecutive months (*p* = 0.526), and a symmetric deformity (*p* = 0.115) with improved outcomes (Table 3).

**Table 3:** The associations between predictor variables and change in CI over course of treatment

Variable	Total (*N* = 72)	ΔCI	*p*
Age			**0.003**
≤ 11	33(45.83%)	7.27 ± 6.87	
> 11	39(54.17%)	1.86 ± 8.05	
Gender			0.151
Male	57(79.17%)	3.20 ± 7.70	
Female	15(20.83%)	8.64 ± 7.70	
Daily usage (hours)			0.058
> 2.5	35(48.61%)	5.54 ± 7.79	
≤ 2.5	37(51.39%)	3.20 ± 8.06	
Time of use (months)			0.526
> 24	40(55.56%)	5.18 ± 8.30	
≤ 24	32(44.44%)	3.28 ± 7.51	
Symmetric deformity			0.115
Symmetry	48(66.67%)	5.01 ± 7.47	
Asymmetry	24(33.33%)	2.99 ± 8.89	

The common side effects were chest pain in 4 (5.6%) patients, swollen skin in 5 (7.0%) patients, chest tightness in 1 (1.4%) patients and erythema in 11 (15.3%) patients (Table 4). The mean follow-up was 3.3 years.

**Table 4:** The side effects of vacuum suction

Side effects	Total (*N* = 72)
Chest pain	4 (5.6%)
Swollen skin	5 (7.0%)
Chest tightness	1 (1.4%)
erythema	11 (15.3%)

## Discussion

The primary feature of PE is the concave deformity of the anterior chest wall. As the condition worsens, the volume of the chest cavity decreases, which has a negative impact on the patient's respiratory system. Severe cases can lead to respiratory infections or obstructive lung disease. The condition is generally congenital and tends to worsen with age, particularly during puberty. The psychological impact of the disease should not be overlooked as it can cause mental health issues such as depression, social withdrawal, and other social problems. The pathogenesis of PE is not yet fully understood, but it may be related to abnormal development of the ribs, sternum, and diaphragmatic central tendon.

Although the Nuss procedure is still the first choice for the surgical intervention of pectus excavatum, that complications may include pneumothorax, mediastinal shift, pericardial injury, correction bar displacement, and the potential for severe postoperative pain and recurrence [12].

Non-invasive methods are available for treating pectus excavatum, and among them, vacuum bells have been continuously developed and enhanced in recent years as a viable option for treatment. In 1910, Lange utilized a vacuum suction device to lift the sunken sternum, but due to material limitations, the application of negative pressure vacuum bells had not gained widespread popularity [23]. However, in 1992, Klobe improved the vacuum bell by using silicone material. Haecker and other researchers then applied the vacuum bell to clinical practice and established multiple models [15]. Recently, aided by advancements in 3D printing, some researchers have employed this technology to craft personalized vacuum bells that match the distinctive chest wall deformities of individual patients [18].

During the course of vacuum bell treatment, patients may experience adverse reactions such as skin bruises, pain, breathing difficulties, and local skin allergies. However, all of these adverse reactions are usually temporary and will disappear shortly after treatment is discontinued. The major advantage of vacuum bells over surgery is that it is a non-invasive treatment with fewer complications and less discomfort during the treatment process. Nevertheless, patients and their families are required to have good compliance and maintain long-term persistence. For most patients who choose vacuum bell therapy, at least 4 h of treatment per day is required for a duration of 1 year or longer in order to achieve stable results. Many of our patients were unable to sustain such a level of commitment. In fact, nearly a quarter of our patients were either lost to follow-up or discontinued vacuum bell use. When combining these figures to assess overall success rates, only 18.36% of patients demonstrated excellent correction in HI, and 9.18% achieved a CI reduction below 10 overall.

The predominant approach was to employ chest wall depth as the evaluative measure to assess the effectiveness of vacuum bell therapy for pectus excavatum. In the initial North American study by Obermeyer et al., involving 115 patients with a median follow-up of 12 months, they reported 20% excellent correction and 17% good correction. Factors predicting excellent correction included initial age under 11 years, initial chest wall depth less than 1.5 cm, vacuum use over 12 consecutive months, and chest wall flexibility [22]. A similar approach by Toselli et al. with 186 patients demonstrated that 35% achieved good or excellent results. Key thresholds for success were an initial pectus depth of 1.8 cm or less and a treatment duration exceeding 12 months [24]. It's worth noting that Haeker et al. showed, in a study involving 140 patients with an average initial deformity depth of 2.7 cm, that 43.6% achieved a fully corrected sternum following a prescribed protocol. These data may represent optimal outcomes for vacuum bell therapy [25].

Furthermore, some studies have delved into changes in the Haller Index during vacuum bell therapy. In Etienne et al.’s study, 31 patients with a median age of 14 underwent vacuum bell therapy, with a median Haller Index of 3.9 before treatment and an average decrease of 0.3 at the latest follow-up. They also noted a more significant improvement in the Haller Index associated with a younger age at treatment initiation [26]. Additionally, in the study by Shigeyuki et al. involving 15 patients, changes in depth and HI did not align. They proposed that the primary factor contributing to the improvement in depth was the thickening of subcutaneous fat [27].

We employed the HI and CI as evaluation criterion for non-surgical treatment of pectus excavatum, which offers the following characteristic: Using imaging metrics as assessment metric provides a standardized and reproducible approach; Depression depth is subject to changes with exercise and weight alteration. In the context of the correction formula, a baseline HI of 3.25 was chosen. Patients whose HI decreased below 3.25 after treatment were regarded as achieving excellent outcomes, thereby potentially reducing the necessity for surgical intervention.

When utilizing HI as a criterion for analysis, vacuum bell therapy enhances chest contour in nearly a quarter of patients, resulting in their Haller Index falling below surgical standards. Our findings revealed that variables associated with “non-poor” outcomes (including results such as excellent, good, and fair.) included an initial age ≤ 11 and a treatment duration ≥ 24 months. Initial HI, symmetric deformity, and daily usage time are not statistically significant in a multifactorial logistic regression analysis. the chest wall stiffness and skeletal maturity associated with age may play a more pivotal role in the effectiveness of vacuum bell therapy. Despite 66.7% of patients exhibited symmetrical deformities, the impact of this symmetry on treatment outcomes remains inconclusive. Another potential explanation could be that the earlier study was retrospective and failed to consider confounding factors, possibly biasing the results. The subjective nature of daily usage time as a statistical metric might account for the lack of discernible differences in outcomes.

Results similar to the HI index were observed, demonstrating a correlation between CI variations and the age of PE patients. Patients under the age of 11 exhibited a more favorable correction outcome. The CI demonstrates a higher discriminatory power than the HI in distinguishing between individuals with and without pectus excavatum, clearly delineating the presence of the condition [14]. In this study, the CI has demonstrated potential similar to that of the HI in prognostic research for pectus excavatum.

Among the 72 patients, 9 experienced postoperative recurrence after Nuss surgery, with 3 of 9 achieving “non-poor” therapeutic efficacy. Most complications during vacuum bell treatment are minor, such as spotting and erythema, and usually require only brief discontinuation before regular use can be resumed.

Based on our findings, physicians can consider providing vacuum bell therapy to pediatric patients below the age of 11. In cases where inadequate correction persists after 24 months, patients still fall within the optimal age range for Nuss surgery can still undergo operation [12]. For patients whose age surpasses the recommended range for Nuss surgery, personalized intensified treatment approaches were implemented. It is important to acknowledge that many patients may struggle with maintaining consistent usage, and ensuring treatment compliance becomes a significant consideration.

The limitations of this study include its retrospective design, as well as the exclusion of certain patients from the statistical analysis due to loss to follow-up, non-adherence to treatment, or insufficient data availability. The subjective nature of daily vacuum bell duration and pressure, as reported by patients, may introduce variability influenced by factors such as patient activities, chronological age, skeletal maturation, and subsequent alterations in applied vacuum bell pressure. Furthermore, logistic regression analysis may be susceptible to confounding factors, including variations in vacuum bell types and models. Additionally, undergoing chest CT scans exposes patients to X-rays which raises particular concerns for adolescents due to their heightened tissue and cellular sensitivity, possibly leading to inherent radiation-related risks.

## Conclusions

According to our proposed assessment criteria utilizing the HI index and CI index, vacuum bell therapy is a potential non-surgical intervention for pectus excavatum. When assessing HI, 18 patients (25.0%) showed excellent correction, and 9 patients (12.5%) achieved a CI reduction below 10. Variables predicting better outcome including initial age ≤ 11 years both in HI and CI and vacuum bell use over 24 consecutive months in HI, offering valuable guidance for subsequent vacuum bell therapies in pectus excavatum management. However, further prospective investigations are necessary to substantiate these findings.

### Supplementary Information

**Supplementary Material 1.**

## Data Availability

The datasets used and/or analyzed during the current study are available from the corresponding author on reasonable request.

## Abbreviations

CT: Computed tomography
HI: The haller index
CI: The correction index
PE: Pectus excavatum
OR: Odds ratio
